# The Role of Parenting Behaviors and Their Influence on Adolescent Drunk and Drugged Driving: 2016–2019, USA

**DOI:** 10.3390/ijerph21060695

**Published:** 2024-05-28

**Authors:** R. Andrew Yockey, Cristina S. Barroso, Rachel A. Hoopsick

**Affiliations:** 1 Department of Biostatistics and Epidemiology, School of Public Health, University of North Texas Health Science Center, Fort Worth, TX 76109, USA; 2 Department of Internal Medicine and Geriatrics, Texas College of Osteopathic Medicine, University of North Texas Health Science Center, Fort Worth, TX 76109, USA; 3 School of Public Health San Antonio, University of Texas, San Antonio, TX 78229, USA; barroso@uthscsa.edu; 4 Department of Kinesiology and Community Health, College of Applied Health Sciences, University of Illinois Urbana-Champaign, Champaign, IL 61820, USA; hoopsick@illinois.edu

**Keywords:** driving, risk behavior, parenting, drugged driving

## Abstract

Drugged driving, the act of driving a vehicle under the influence of illicit drugs, by adolescents is a serious public health concern. Many factors contribute to this risk behavior, but much less is known regarding the role of parenting behaviors in this phenomenon. The purpose of this study was to examine specific parenting behaviors and their influence among a nationally representative sample of adolescents. Pooled data from the 2016–2019 National Survey on Drug Use and Health (NSDUH) among 17,520 adolescents ages 16–17 years old were analyzed. Differences were found in specific parenting behaviors and adolescent drugged/drunk driving, with parents not checking homework and not telling their children they are proud of them being the most influential. Findings from the present study may inform drugged driving prevention programs for parents and adolescents and enhance road safety interventions.

## 1. Introduction

Motor vehicle crashes are the leading cause of death in adolescents in the United States (US), with an estimated 2800 fatalities and hundreds of thousands of injuries recorded in 2020 [1]. Moreover, the exorbitant estimated costs of treatment, emergency department visits, and hospitalizations [2,3] related to motor vehicle crashes total billions of dollars, straining healthcare organizations and systems. Regarding driving, adolescents are more likely to not wear seatbelts and speed over the legal limit [4], making them a priority population for intervention and behavioral change.

The use of alcohol and other drugs while driving, known as “drugged driving”, is a major risk factor for these crashes among adolescents. Data from the 2019 Youth Risk Behavior Surveillance System estimated that 5.4% of high school students drove under the influence of alcohol in the past 30 days [5]. In addition, more than 1 in 10 adolescents (16.7%) rode with a driver who had been drinking alcohol. Recent research from Yockey and Barroso estimated that among a large national sample of adolescents in the US, 2.0% of adolescents drove under the influence of alcohol in the past year, 5.7% drove under the influence of marijuana in the past year, and 0.5% of adolescents drove under the influence of other drugs other than marijuana in the past year [6] The use of drugs while operating vehicles is a known risk factor for increased crashes and fatalities [5], placing adolescents at highest risk for injury compared to other age groups.

The available evidence on adolescent drugged driving points towards several risk factors at the individual, psychological, and psychosocial levels. One factor that remains to be explored is the influence of parenting styles. Parenting styles encompass a variety of behaviors and beliefs and are known to influence adolescents’ psychosocial and developmental trajectories [7]. Proposed by Baumrind, three parenting styles (i.e., “authoritative” –high demandingness, high response; “authoritarian”–low in response, high in demandingness; and “permissive”–high responsiveness, low demandingness) have a profound effect on adolescent psychosocial functioning [7]. These parenting styles are known to affect adolescent development and engagement in risky behaviors (e.g., substance use, risky driving) [8]. Indeed, early evidence points towards certain parenting styles having an effect on adolescent driving practices–namely, that authoritative parenting styles (e.g., parental restrictions, monitoring) are associated with adolescent risky driving [9]. Another study examining parenting styles and adolescent driving behaviors using the National Young Driver Survey found that teens with authoritarian or authoritative parents reported greater use of seatbelts and lower risk of speeding [10].

Previous research has shown that specific parenting styles (e.g., low monitoring) might protect against driving under the influence or riding with someone under the influence [11]. However, few studies have examined the influence of parenting behaviors on adolescent drugged and drunk driving using national data. Research is warranted to inform prevention efforts and policy initiatives to promote adolescent health and well-being. Using nationally representative data, the purpose of this brief is to examine the influence of parenting behaviors and their influence on adolescent drunk and drugged driving over multiple years of data.

## 2. Methods

We used data from the 2016–2019 National Survey on Drug Use and Health (NSDUH). The NSDUH is a cross-sectional, annual, nationally representative survey conducted in the United States and the District of Columbia to assess behavioral health utilization, substance use, and mental health among individuals 12 years or older. The NSDUH uses a complex sampling design to ensure adequate representation of participants. To ensure adequacy and privacy of responses, the NSDUH uses computer-assisted interviewing (CAI) techniques for all participants. Other details of the NSDUH can be found elsewhere [12]. We pooled data from 2016 to 2019, as this was the first year that assessed driving under the influence of marijuana. Response rates were >70% for each wave examined in this study. Our sample was limited to 16–17-year-olds, as this is the average age for licensure in most states.

## 3. Measures

### 3.1. Driving under the Influence of Alcohol, Marijuana, and Other Drugs

Past-year driving under the influence of alcohol was assessed by the question: “Did you drive a car or vehicle while under the influence of alcohol in the past-12 months?” Past-year driving under the influence of marijuana was assessed by the question: “Did you drive a car or vehicle while under the influence of marijuana in the past-12 months?” Prior year driving under the influence of other drugs (other than marijuana) was assessed by the question: “Did you drive a car or vehicle while under the influence of cocaine, inhalants, methamphetamine, heroin, or hallucinogens?” Responses were binary in nature (1 = “Yes”, 0 = “No”).

### 3.2. Parenting Behaviors

Parenting behavior questions asked adolescents to report how often their parent(s) engaged in the following authoritative parenting behaviors in the past 12 months: (1) checked to see if their homework was done, (2) helped them with homework, (3) were told to do chores around the house, (4) limited the amount of TV watched, (5) limited the amount of time spent out on a school night, (6) told them they did a good job, and (7) told them they were proud of them. All questions had a four-point scale response option (1 = never, 2 = seldom, 3 = sometimes, 4 = always). We dichotomized response options into “Never/Seldom” and “Sometimes/Always”.

### 3.3. Covariates

We controlled for the potential confounding effects of participants’ biological sex (Male, Female), Race (Non-Hispanic White, Non-Hispanic African American, Hispanic, and Other/Mixed Race), and Metro Status (Metro, Non-Metro, and Rural). Here, “Other/Mixed Race” is a combination of Native American, Native Hawaiian/Pacific Islander, and Multi-Racial, due to small cell sizes.

## 4. Analysis Plan

We used multiple-imputed variables provided by NSDUH to limit the amount of missing data. We opted to do a complete case analysis since data were missing <5% on all variables, and these methods would not bias estimates [13]. Frequencies with 95% confidence intervals were estimated to capture participant demographics and the prevalence of drugged driving and driving under the influence of alcohol. Multivariable logistic regression analyses were built to determine conditional associations between driving under the influence of alcohol and drugged driving. Variance inflation factors were <3.0, indicating multicollinearity was not a problem. We estimated prevalence ratios with a log-linear link using a Poisson distribution [14], given that prevalence ratios are easier to interpret than odds ratios [14]. All analyses were conducted in Stata (v. 17.0) using the ‘svy’ commands and were weighted to account for the complex sampling design and non-response [12]. The level of significance was set at *p* < 0.05.

## 5. Results

### Demographic Characteristics and Prevalence of Drugged Driving Behaviors

The analytic sample consisted of 16,840 adolescents aged 16–17-years old (See Table 1). The sample consisted of equal percentages of males and females (50.0 vs. 50.0%, respectively). Overall, an estimated 1.96% of adolescents drove under the influence of alcohol, 5.57% drove under the influence of marijuana, and 0.48% drove under the influence of other drugs other than marijuana in the past year.

## 6. Final Multivariate Models

### 6.1. Driving under the Influence of Alcohol

There were no differences between males and females on past-year driving under the influence of alcohol (*p* = 0.50) (See Table 2). Compared to adolescents who were White, adolescents who were African American (aPR: 0.36, 95% CI 0.20, 0.65), Hispanic (aPR: 0.54, 95% CI 0.37, 0.79), and Other/Mixed Race (aPR: 0.60, 95% CI 0.37, 0.96) were less likely to drive under the influence of alcohol. No differences were found between rurality status. A parenting factor that placed adolescents at risk for driving under the influence of alcohol was parents never/seldom checking homework (aPR: 1.55, 95% CI 1.15, 2.07). Adolescents whose parents limited their time out on a school night were less likely to drive under the influence of alcohol (aPR: 0.68, 95% CI 0.51, 0.90).

### 6.2. Driving under the Influence of Marijuana

There were no differences between males and females. Compared to adolescents who were White, adolescents who were African American (aPR: 0.51, 95% CI 0.38, 0.68), Other/Mixed Race (aPR: 0.63, 95% CI 0.48, 0.83), and Hispanic (aPR: 0.65, 95% CI 0.51, 0.82) were less likely to drive under the influence of marijuana. Significant parenting factors that placed adolescents at risk for driving under the influence of marijuana were parents seldom/never checking homework (aPR: 1.48, 95% CI 1.23, 1.78), limiting their TV time (aPR: 2.04, 95% CI 1.62, 2.56), and never/seldom telling their children that they did a good job (aPR: 1.35, 95% CI 1.05, 1.73). Adolescents were less likely to drive under the influence of marijuana if their parents never/seldom limited their time out on a school night (aPR: 0.81, 95% CI 0.68, 0.96).

### 6.3. Driving under the Influence of Other Drugs Other than Marijuana

There was only one significant parenting factor contributing to a higher risk of driving under the influence of drugs other than marijuana–namely, adolescents were 2.13 (95% CI 1.09, 4.21) times more likely to engage in this behavior if parents never/seldom checked homework.

## 7. Discussion

### 7.1. Principal Findings

This is one of the first studies examining the influence of parenting behaviors on adolescent drunk and drugged driving using a large national sample. Findings from the present study indicate that specific parenting behaviors might increase and decrease the risk for adolescent drunk and drugged driving. Specifically, never/seldom checking homework, limiting TV time, and parents not telling their children they are proud of them were associated with an increased risk for drunk and drugged driving. Differences were also found based on race/ethnicity.

### 7.2. Findings in Context

The impact of parenting styles and practices on adolescent development influences several developmental trajectories, including psychosocial, biological, and environmental [15]. Moreover, the influence of parenting practices at an early developmental age can have long-term benefits and consequences on the developing adolescent. Our results are some of the first highlighting the impact of parenting behaviors on adolescent drunk and drugged driving. Specifically, adolescents were at greater risk for drunk and drugged driving if their homework was not checked by a parent. Homework is viewed by several scholars [16] as an activity linking the connection between parent and child, understanding their child, and strengthening the bond with the child. Previous research demonstrates the positive benefits of being connected with the parent and increased connection to the school. In one study [17], greater connections to the parent and school environment were associated with fewer risky behaviors (e.g., drug use, delinquency), which may explain our findings. That is, engagement in drunk and drugged driving may be viewed as a mechanism to cope with the lack of bonding they have with their parents. Moreover, this could be seen as ‘neglectful parenting’, a style proposed after Baumrind that emphasizes low response and low demandingness [18]. Research indicates that neglectful parenting leads to worse psychosocial outcomes for the developing adolescent and a higher propensity to engage in risky behaviors [19].

Our results also highlight that other parenting behaviors (e.g., limiting TV time, parents not saying they are proud of them, limiting time out on a school night) are indicative of increased/decreased engagement in drunk and drugged driving. Overall, these behaviors and others are a set of phenomena that can have a profound impact on the typical developing adolescent [7,8]. More research is warranted on the relationship between parenting behaviors and adolescent risk behavior engagement, but several practices that parents may implement to reduce the development of risk behaviors include increasing the self-esteem of their child, setting limits with their child, and increasing communication and bonding/social time with their child [20]. For example, increasing bonding/social time with the child by enhancing communication, giving them praise, and helping them with stressful aspects of life leads to lower risk-taking behavior and lower psychopathology [21].

In the context of driving, parents can set limits on driving for their child (e.g., no driving at night) and become more involved in every step of the licensure process. For example, one study found that setting limits on driving to ensure the safety of their child reduced risky driving behavior [9], while another study found that strict limits on driving to ensure safety resulted in fewer crashes and traffic violations [22].

### 7.3. Strengths and Limitations

The use of a large sample of adolescents enhances the robustness of our analyses. Several limitations should be noted, however. The NSDUH is a cross-sectional designed study, thus limiting our ability to infer causality. The NSDUH does not ask about the mode of marijuana use (e.g., edible, vape) or alcohol use; future research is warranted on the different modes of cannabis/alcohol use and their respective impact. Additionally, validated scales for parenting behaviors are warranted to be implemented in practice. 

## 8. Conclusions

Drunk and drugged driving among adolescents are critical public health concerns. Our study is one of the first to examine parenting behaviors and their impact on adolescent drunk and drugged driving using a national sample. Findings from the present study can be used to inform drugged driving prevention programs for parents and adolescents as well as enhance road safety interventions.

## Figures and Tables

**Table 1:** Demographics (N = 16,840).

Variable	% [95% CI]
Biological Sex	
Male	50.0 [48.9, 50.9]
Female	50.0 [49.1, 51.0]
Race/Ethnicity	
Non-Hispanic White	53.4 [52.4, 54.4]
Non-Hispanic Black/African American	13.3 [12.6, 13.9]
Hispanic	23.8 [22.9, 24.7]
Other/Mixed Race	9.52 [8.96, 10.1]
Metropolitan Status	
Large Metro	57.2 [56.2, 58.1]
Small Metro	29.2 [28.3, 30.0]
Nonmetro	13.7 [13.1, 14.3]

**Table 2:** Conditional associations towards driving under the influence of alcohol, marijuana, and other drugs (other than marijuana).

Variable	Driving Under the Influence of Alcohol (*n* = 388)	Driving Under the Influence of Marijuana (*n* = 1031)	Driving Under the Influence of Drugs other than Marijuana (*n* = 87)
Biological Sex			
Male	1.00	1.00	1.00
Female	1.10 [0.84, 1.43]	0.86 [0.74, 1.01]	1.03 [0.59, 1.79]
Race/Ethnicity			
Non-Hispanic White	1.00	1.00	1.00
Non-Hispanic Black/African American	0.36 [0.20, 0.65] *	0.51 [0.38, 0.68] *	0.38 [0.08, 1.77]
Hispanic	0.54 [0.37, 0.79] *	0.65 [0.51, 0.82] *	0.51 [0.21, 1.24]
Other/Mixed	0.60 [0.37, 0.96] *	0.63 [0.48, 0.83] *	1.04 [0.44, 2.44]
County Status			
Large Metro	1.00	1.00	1.00
Small Metro	1.29 [0.95, 1.75]	1.19 [1.00, 1.41]	1.53 [0.77, 3.05]
Non-Metro	1.29 [0.93, 1.80]	0.90 [0.72, 1.12]	1.71 [0.78, 3.74]
Parenting Behaviors			
Parent Checked to see if Homework was done			
Sometimes/Always	1.00	1.00	1.00
Never/Seldom	1.55 [1.15, 2.07] *	1.48 [1.23, 1.78] *	2.13 [1.09, 4.21] *
Parents Helped you with Homework			
Sometimes/Always	1.00	1.00	1.00
Never/Seldom	1.26 [0.92, 1.71]	1.12 [0.93, 1.36]	1.78 [0.92, 3.43]
Parents Told You to Help with Chores			
Sometimes/Always	1.00	1.00	1.00
Never/Seldom	1.40 [0.98, 1.99]	1.22 [0.99, 1.51]	1.73 [0.94, 3.18]
Parents Limited TV Time			
Sometimes/Always	1.00	1.00	1.00
Never/Seldom	1.34 [0.93, 1.94]	2.04 [1.62, 2.56] *	0.67 [0.31, 1.46]
Parents Limit Time Out on a School Night			
Sometimes/Always	1.00	1.00	1.00
Never/Seldom	0.68 [0.51, 0.91] *	0.81 [0.68, 0.96] *	0.75 [0.41, 1.38]
Parents Told You That you did a Good Job			
Sometimes/Always	1.00	1.00	1.00
Never/Seldom	1.38 [0.93, 2.05]	1.35 [1.05, 1.73] *	1.46 [0.75, 2.83]
Parents Told You that They are Proud of You			
Sometimes/Always	1.00	1.00	1.00
Never/Seldom	1.29 [0.86, 1.95]	1.11 [0.85, 1.45]	2.01 [0.90, 4.47]

* Bolded values indicate a level of significance of *p* < 0.05.

## Data Availability

The data presented in this study are from the 2016–2019 National Survey on Drug Use and Health (NSDUH).
